# Association between anxiety symptoms and atrial fibrillation in a community cohort of Chinese older adults: a case-control study

**DOI:** 10.1186/s12872-021-02278-x

**Published:** 2021-09-30

**Authors:** Zhu-Xia Shen, Yu-Min Sun, Hui-Hui Gu, Yan Zhang, Zhi-Wen Shen, Xiao-Niu Liang, Ding Ding, Jun Wang

**Affiliations:** 1 Department of Cardiology, Jing’an District Centre Hospital of Shanghai, Fudan University, 259 Xikang Rd, Shanghai, 200040 China; 2 Department of Neurology, Huashan Hospital, WHO Collaborating Center for Research and Training in Neurosciences, Fudan University, Shanghai, China; 3 National Clinical Research Center for Aging and Medicine, Huashan Hospital, Fudan University, Shanghai, China

**Keywords:** Anxiety disorders, Atrial fibrillation, Cardiac epidemiology, Risk management, Geriatric medicine

## Abstract

**Background:**

The association between anxiety and atrial fibrillation (AF) remains unclear. Moreover, this association has rarely been studied in Chinese individuals aged 60 years or older. This study investigated the association between anxiety and AF in a community-based case-control study of older adult residents in urban China.

**Methods:**

The cases and controls were from a community-based study conducted in the Jingansi community in Shanghai, China, between January 2010 and December 2012. A total of 3622 residents aged 60 years or older without severe vision, hearing, or speaking impairments were eligible to participate in the physical examinations and questionnaire survey. AF was assessed based on a previous physician’s diagnosis, electrocardiogram, ambulatory electrocardiogram, or echocardiogram. Anxiety was evaluated using the Zung Self-Rating Anxiety Scale (ZSAS). Using the AF group as a reference, the control group consisted of randomly selected age- and sex-matched individuals in a 1:5 ratio (case:control = 1:5). The association between anxiety and AF in the AF group and the multifactor-matched control group was explored using logistic regression.

**Results:**

In the AF and control groups, after adjusting for a history of coronary heart disease, valvular heart disease, hypertension, stroke, hyperlipidemia, and diabetes, as well as depression score, ZSAS scores (odds ratio 1.07; 95% confidence interval 1.02–1.12; p = 0.003), and anxiety symptoms (odds ratio 3.94; 95% confidence interval 1.06–14.70; *p* = 0.041) were associated with AF.

**Conclusions:**

Anxiety symptoms were associated with AF in a Chinese older population. This suggests that older adults who have anxiety symptoms may need psychological intervention or treatment in daily life and care.

## Background

Increased morbidity and mortality rates are found among patients with atrial fibrillation (AF), the most common type of arrhythmia [1–3]. AF can cause a five-fold, two-fold, three-fold, and two-fold increase in the risk of stroke, dementia, heart failure, and myocardial infarction, respectively [4, 5]. AF can increase the risk of overall mortality by 40–90% [5].

The prevalence of AF increases with increasing age. The estimated prevalence of AF in China, Australia, the United States, and Europe has been shown to increase from 1 to 4% in adults to more than 8.8% in older adults over 80 years of age [3, 4]. Among older adults aged 60 years or older in California, USA, the prevalence of AF was significantly higher in Caucasians (8.0%) than in African–Americans (3.8%), Hispanics (3.6%), and Asians (3.9%) [6]. China is a low-risk region for AF [3]. Previous epidemiological studies have reported risk factors for AF such as aging, obesity, hypertension, diabetes, tobacco smoking, and alcohol consumption [5, 7].

Anxiety is a common mental health problem characterized by negative anticipation of future threats and accompanied by a feeling of restlessness and/or somatic symptoms of tension [8]. When the feeling is excessive and uncontrolled, anxiety becomes pathological [8, 9]. Anxiety disorders are associated with substantial functional impairment [9]. The prevalence of anxiety disorders is estimated to be 2.0% (95% confidence interval (CI) 1.1–3.2 %) among Chinese male adults and 3.3 % (95% CI 1.6–5.3 %) among Chinese female adults [10]. A meta-analysis concluded that anxiety symptoms were associated with an elevated risk of cardiovascular events, including stroke (relative risk [RR] 1.71, 95% CI 1.18–2.50), coronary heart disease (CHD; RR 1.41, 95% CI 1.23–1.61), heart failure (RR 1.35, 95% CI 1.11–1.64), and cardiovascular death (RR 1.41, 95% CI 1.13–1.76) [11]. Anxiety was associated with AF in hospitalized patients [12, 13], and a prospective study showed that panic disorder (a common type of anxiety disorder) was a risk factor for AF in a Chinese population [14]. However, 3 prospective studies failed to find evidence that anxiety was a risk factor for AF in a Caucasian population in the United States or Norway [11, 15–17].

This study aimed to assess the association between anxiety and AF among community-dwelling older adults in urban Shanghai, China.

## Methods

### Recruitment of participants

Residents aged 60 years or older from the Jingansi community in Shanghai, China, were enrolled in the study. The enrollment period started in January 2010 and ended in December 2012. The detailed participant recruitment protocol of the Shanghai Aging Study has been published previously [18]. All participants were recruited from the Jingansi community. Eligible participants were (1) registered residents of the Jingansi community; (2) at least 60 years old; (3) individuals without severe vision, hearing, or speaking impairments; and (4) able to communicate and participate in physical examinations and a questionnaire survey. A total of 3622 participants were included in our study.

**Fig. 1:**
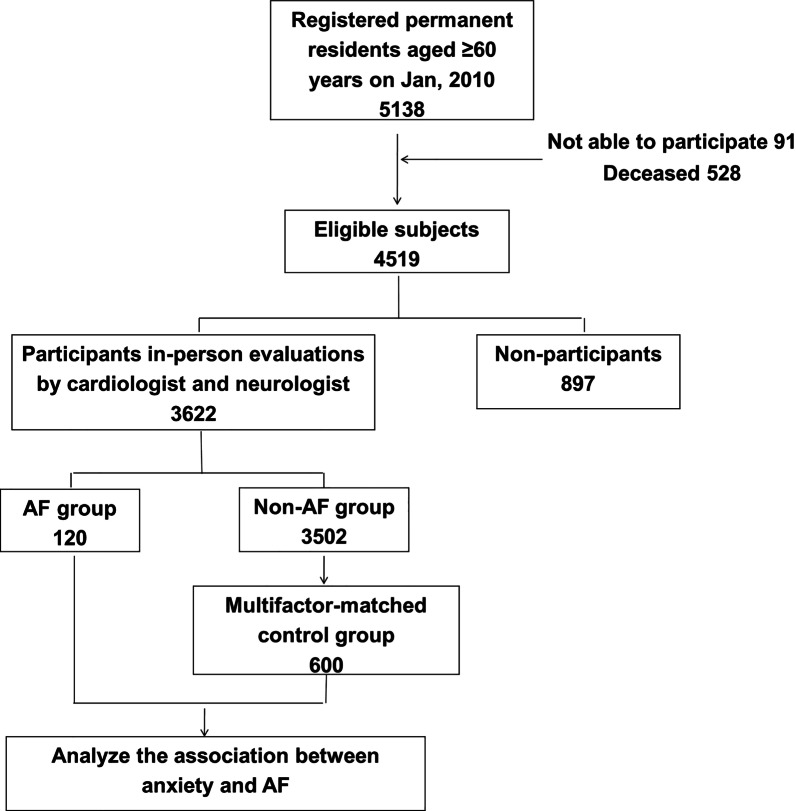
Flow diagram of AF (cases) and controls (multifactor-matched controls without AF) for the study. AF, atrial fibrillation

The selection of cases and controls is summarized in the flowchart (Fig. 1). For cases, each participant was examined by a cardiologist to diagnose AF. First, each participant was examined with an electrocardiogram (ECG) and asked about his or her AF history. Participants with AF history were asked which type of AF was diagnosed: paroxysmal AF, persistent AF, or permanent AF. Second, participants with palpitations or chest distress but without AF history underwent ambulatory ECG (Holter) or echocardiogram. AF was defined as (1) self-reported physician-diagnosed AF confirmed by medical records, (2) ECG characteristics (irregular RR intervals with no discernible or distinct P-waves persisting for ≥ 30 s), (3) Holter characteristics (irregular RR intervals with no discernible or distinct P-waves persisting for ≥ 30 s), or (4) echocardiogram characteristics (atrial rate more than 350 beats per minute, irregular ventricular rhythm, and blood flow of mitral valve represented as a single peak). To further clarify that age was not a confounder between AF and ZSAS scores, we randomly selected a multifactor-matched control group that was age- and sex-matched with the AF patients group (case: control = 1: 5) to verify the association between anxiety and AF.

## Demographic characteristics of participants

At baseline, demographic factors, lifestyle characteristics, and medical histories of the participants were collected via face-to-face interviews by trained interviewers (nurses and cardiologists) using interviewer-administered questionnaires and physical examinations. Each participant was examined by a cardiologist to diagnose AF and by a neurologist to evaluate anxiety symptoms on the same day. Personal information, including birth date, sex, education, and cigarette smoking and alcohol consumption habits, were obtained from each participant. Additionally, a physical examination was conducted to collect anthropometric data, including weight and height. Blood was collected from the majority (91 %) of participants for laboratory tests at baseline to determine high-density lipoprotein cholesterol (HDL-C) levels in plasma. Body mass index (BMI, kg/m^2^) was calculated as weight divided by the squared height. A comprehensive medical history of physician-diagnosed diseases such as diabetes, hypertension, hyperlipidemia, stroke, coronary artery disease, and valvular heart disease was collected and confirmed from patients’ medical records. Depression was measured using the Center for Epidemiologic Studies Depression Scale (CES-D) questionnaire. Depression was defined by a total score ≥ 16. Detailed definitions of these comorbid conditions have been provided in previously published reports [18–22].

## Evaluation of anxiety symptoms

Anxiety was measured using the Zung Self-Rating Anxiety Scale (ZSAS) [23] for each participant in this study. The ZSAS, which includes questions on psychological, affective, and somatic symptoms, evaluates the frequency of anxiety symptoms in the previous 4 weeks on a 4-point scale. The scale comprises 20 items (15 negative and 5 positive items) for evaluating anxiety-associated symptoms. Each item is scored on a scale of 1 to 4 as follows: 1 = never or rarely; 2 = some of the time; 3 = frequently; and 4 = most or all of the time. The ZSAS score was obtained by summing the points of the 20 items; the minimum is 20 and the maximum is 80, with higher scores indicating more severe anxiety symptoms. Anxiety is defined by a ZSAS score > 44.

### Statistical analysis

Continuous variables are expressed as means ± standard deviations, and categorical variables are expressed as frequencies (%). Student’s t-test and Pearson’s chi-squared test were used to compare continuous variables and categorical variables, respectively. A univariate logistic regression model was used to detect the association between AF and its potential risk factors.

Considering other potential confounders, multivariate logistic regression was conducted between AF and ZSAS scores after adjustment for CHD, valvular heart disease, hypertension, stroke, hyperlipidemia, diabetes, and depression score. The risk and strength of association are presented in terms of odds ratios (ORs) and 95 % confidence intervals (CIs).

All *p*-values and 95 % CIs were estimated using 2-tailed tests. Differences were considered to be statistically significant at *p* < 0.05. Data were analyzed using R3.3.3 and SPSS19 software.

## Results

A total of 3622 participants were enrolled in this study, and 720 were included in the final analysis: 120 patients with AF (cases) and 600 controls (Fig. 1). The baseline demographic characteristics of cases and controls are shown in Table 1. There were no differences in age, sex, BMI, years of education, HDL-C, cigarette smoking, alcohol consumption, history of diabetes, and hyperlipidemia between the AF and control groups (Table 1). The average ZSAS scores of the AF and control groups were 29.3 ± 7.5 and 26.7 ± 6.0, respectively. Compared with the control group, patients in the AF group had higher ZSAS scores (*p* < 0.001), anxiety rates (*p* = 0.011), and CES-D scores (*p* = 0.055). In addition, the prevalence rates of CHD (*p* < 0.001), valvular heart disease (*p* = 0.002), hypertension (*p* = 0.017), and stroke (*p* = 0.004) were higher in the AF group.

**Table 1:** Demographic factors, anthropometric indexes, lifestyle characteristics, and medical history in cases and controls

	AF (n = 120)	Controls (n = 600)	*p* value^b^
Age, years	76.3 ± 7.4	76.3 ± 7.4	0.981
Male, n (%)	54 (45.0)	270 (45.0)	1
BMI, kg/m^2^	24.2 ± 3.5	24.1 ± 3.6	0.774
Education, years	11.3 ± 4.9	11.0 ± 5.0	0.637
HDL-C, mmol/L	1.36 ± 0.40	1.36 ± 0.34	0.980
Cigarette smoking, n (%)	9 (7.5)	48 (8.0)	0.861
Alcohol consumption, n (%)	8 (6.7)	44 (7.3)	0.793
Anxiety (ZSAS > 44)	6 (5.0)	7 (1.2)	0.011^a^
ZSAS score	29.3 ± 7.5	26.7 ± 6.0	< 0.001
CES-D score	10.1 ± 9.2	8.5 ± 7.9	0.055
History of disease			
CHD, n (%)	57 (47.5)	102 (17.0)	< 0.001
VHD, n (%)	6 (5.0)	4 (0.7)	0.002^a^
Diabetes, n (%)	18 (15.0)	95 (15.8)	0.797
Hypertension, n (%)	83 (69.2)	343 (57.2)	0.017
Hyperlipidemia, n (%)	40 (33.3)	194 (32.3)	0.867
Stroke, n (%)	34 (28.3)	103 (17.2)	0.004

^a^Fisher’s exact test for count data; ^b^comparison between AF group and controls; *p* value of Student’s t-test or Chi-square test between the 2 groups. BMI, body mass index; CHD, coronary heart disease; HDL-C, high-density lipoprotein cholesterol; VHD, valvular heart disease; ZSAS, Zung Self-Rating Anxiety Scale; CES-D, Center for Epidemiologic Studies Depression Scale

As shown in Table 2, univariate logistic regression indicated that increased ZSAS scores (OR 1.06; 95% CI 1.03–1.09; *p* < 0.001) and anxiety (OR 4.53; 95% CI 1.49–13.71; *p* = 0.008) were associated with AF. A multivariate logistic regression model indicated that ZSAS scores and anxiety were positively associated with AF after adjustment for history of CHD, valvular heart disease, hypertension, stroke, hyperlipidemia, and diabetes, as well as depression score. A 1-unit increase in ZSAS score was associated with a 7 % increased risk of AF (OR 1.07; 95% CI 1.02–1.12; *p* = 0.003). Anxiety was associated with a 3.94-fold increase in AF risk (OR 3.94; 95% CI 1.06–14.70; *p* = 0.041).

**Table 2:** Association between AF and multiple factors, including ZSAS score, in logistic regression models in cases and controls

	Model 1		Model 2		Model 2	
	OR (95 % CI)	*p* value	OR (95 % CI)	*p* value	OR (95 % CI)	*p* value
ZSAS score	1.06 (1.03, 1.09)	< 0.001	1.07 (1.02, 1.12)	0.003		
Anxiety	4.53 (1.49, 13.71)	0.008			3.94 (1.06, 14.70)	0.041
CHD	4.37 (2.88, 6.64)	< 0.001	4.27 (2.67, 6.84)	< 0.001	4.34 (2.72, 6.93)	< 0.001
VHD	7.78 (2.16, 27.99)	0.002	9.24 (2.41, 35.37)	0.001	9.89 (2.60, 37.70)	0.001
Hypertension	1.66 (1.09, 2.53)	0.018	1.19 (0.74, 1.91)	0.47	1.24 (0.78, 1.99)	0.36
Stroke	1.91 (1.22, 2.99)	0.005	1.45 (0.88, 2.38)	0.15	1.47 (0.89, 2.41)	0.13
Hyperlipidemia	1.04 (0.68, 1.57)	0.87	0.77 (0.49, 1.22)	0.26	0.82 (0.52,1.29)	0.38
Diabetes	0.93 (0.54, 1.61)	0.8	0.72 (0.40, 1.31)	0.28	0.72 (0.40,1.30)	0.28
CES-D	1.02 (1.00, 1.05)	0.056	0.97 (0.93, 1.00)	0.07	1.00 (0.97, 1.02)	0.74

Model 1, univariate logistic regression. Model 2, multivariate logistic regression adjusted for CHD, VHD, hypertension, stroke, hyperlipidemia, diabetes and CES-D

## Discussion

This study demonstrated that anxiety was positively associated with AF after adjusting for a history of CHD, valvular heart disease, hypertension, stroke, hyperlipidemia, and diabetes, as well as depression score.

Our findings are consistent with those of previous studies. In a study from the United Kingdom, 38 % of AF patients and 22 % of hypertensive patients had trait anxiety, which was evaluated using the State-Trait Anxiety Inventory at baseline, and AF patients had significantly higher levels of anxiety (*p* < 0.05) [12]. In China, a similar study was conducted in a hospital in Shanghai. The results showed that 53 patients with AF (66.3 %) and 5 control participants (6.25 %) had emotional disorders that were evaluated by the Hospital Anxiety and Depression Scale, where a high proportion of patients with AF had emotional disorders, such as anxiety or depression (*p* < 0.05) [13]. In 42,768 participants from a Chinese population in Taiwan, after adjusting for risk factors and comorbidities, panic disorder (a common anxiety disorder) was independently associated with an increased risk of AF (hazard ratio [HR] 1.73; 95% CI 1.26–2.37) [14]; in that study, there were more female participants (64 %) and the average age (46.3 ± 15.0 years) was relatively younger compared with that of the community-dwelling population in this study. However, some population-based studies have reported contrary results. Among 37,402 adult residents in Norway, anxiety, assessed using the Hospital Anxiety and Depression Scale, was not associated with incident AF after adjusting for risk factors (symptoms of mild to moderate anxiety, HR 1.1, 95% CI 0.9–1.5; symptoms of severe anxiety, HR 1.0, 95% CI 0.8–1.4 ) [17]. Among 30,746 female individuals in the United States, the results of an age-stratified proportional hazards model showed that anxiety, assessed with a 5-item subscale of the Short-Form 36 health status survey, was unrelated to AF risk (HR 0.90, 95% CI 0.47–1.75) [16]. Among 3682 participants of the Framingham Offspring Study, anxiety scores, assessed with an anxiety scale, were not associated with AF in male (RR 1.10; 95% CI 0.95–1.27) or female (RR 1.03; 95% CI 0.81–1.31) participants after adjusting for risk factors [15]. The use of different instruments for assessing anxiety symptoms and the race difference are possible reasons for the results of this study being in contrast with those of the three prospective studies earlier mentioned. Moreover, several previous epidemiological studies have reported that hyperlipidemia and diabetes are risk factors for AF [5]. In our study, there was no significant association between AF and diabetes or hyperlipidemia. The relatively small sample size and the race difference are possible reasons for the different findings.

The autonomic nervous system may be the conduit between AF and anxiety [24]. Autonomic nervous system activation is capable of causing atrial electrophysiology to change in obvious and heterogeneous ways and can induce AF [25]. The postulated mechanisms include cardiac autonomic dysfunction (such as sympathetic predominance and parasympathetic withdrawal) and the instability of cardiac repolarization [5, 26]. Depression, which is related to anxiety [27], also played a part in the process [26]. Cole et al. reported that anxiety led to depression in children and adolescents in a 3-year longitudinal study [27]. Thus, after adjustment for CHD, valvular heart disease, hypertension, stroke, hyperlipidemia, diabetes, and depression score, we analyzed the association between anxiety and AF. The number of individuals with anxiety symptoms (ZSAS score > 44) was 6 (5.0 %) in the AF group and 7 (1.2 %) in the controls. Although the sample size was small, the results showed that anxiety symptoms were related to AF. The results also showed that the ZSAS score was related to AF, even when it was below the threshold of the definition of anxiety (i.e., ZSAS score > 44). It has been suggested that anxiety might be a predictor of a high-risk cardiac substrate that can induce the occurrence of AF; moreover, individuals who had frequent AF might also potentially develop anxiety due to the somatic symptoms related to AF or associated treatments (such as radiofrequency ablation) [26]. Nevertheless, the underlying mechanism of the association between anxiety and AF is unclear and needs to be investigated.

This study has several strengths. First, the diagnosis of AF was accurate and incorporated both current AF and history of AF. AF was defined by self-reporting and the characteristics of ECG, ambulatory ECG, or echocardiogram. Second, detailed medical data and objective health measures were collected. Third, using a multifactor-matched control group, the associations between AF and potential risk factors are reliable. This method may avoid false associations between AF and a history of diseases based on their connection to a common risk factor, namely age.

Some limitations of our study must be considered. First, a causal relationship between anxiety symptoms and AF could not be inferred; follow-up might help understand the connection between the two. Second, our research site was in the center of an urban area of Shanghai: the educational level of this community is higher than most communities in China, and the living conditions in the urban area are better than most areas in China. Therefore, the results may not be largely generalizable.

## Conclusions

The association between anxiety symptoms and AF was explored in our study. Older adults with AF who have anxiety symptoms may need psychological intervention or treatment in daily life and care. Further prospective studies with larger sample sizes are needed to study the causal relationship between anxiety symptoms and AF.

## Data Availability

The datasets used and/or analyzed during the current study are available from the corresponding author on reasonable request.

## Abbreviations

AF: Atrial fibrillation
BMI: Body mass index
CHD: Coronary heart disease
ZSAS: Zung Self-Rating Anxiety Scale
HDL-C: High-density lipoprotein cholesterol
CES-D: Center for Epidemiologic Studies Depression Scale
